# Differential Condensation of Methane Isotopologues Leading to Isotopic Enrichment under Non-equilibrium Gas–Surface Collision Conditions

**DOI:** 10.1021/acs.jpca.1c07826

**Published:** 2021-10-16

**Authors:** Michelle R. Brann, Stephen P. Hansknecht, Xinyou Ma, S. J. Sibener

**Affiliations:** The James Franck Institute and Department of Chemistry, The University of Chicago, 929 E. 57th Street, Chicago, Illinois 60637, United States

## Abstract

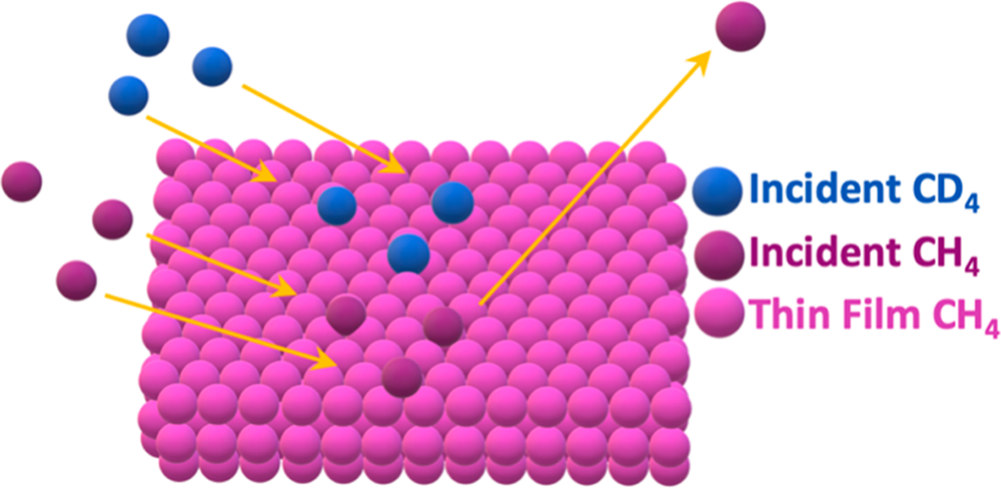

We examine the initial differential sticking probability of CH_4_ and CD_4_ on CH_4_ and CD_4_ ices under nonequilibrium flow conditions using a combination of experimental methods and numerical simulations. The experimental methods include time-resolved *in situ* reflection–absorption infrared spectroscopy (RAIRS) for monitoring on-surface gaseous condensation and complementary King and Wells mass spectrometry techniques for monitoring sticking probabilities that provide confirmatory results via a second independent measurement method. Seeded supersonic beams are employed so that the entrained CH_4_ and CD_4_ have the same incident velocity but different kinetic energies and momenta. We found that as the incident velocity of CH_4_ and CD_4_ increases, the sticking probabilities for both molecules on a CH_4_ condensed film decrease systematically, but that preferential sticking and condensation occur for CD_4_. These observations differ when condensed CD_4_ is used as the target interface, indicating that the film’s phonon and rovibrational densities of states, and collisional energy transfer cross sections, have a role in differential energy accommodation between isotopically substituted incident species. Lastly, we employed a mixed incident supersonic beam composed of both CH_4_ and CD_4_ in a 3:1 ratio and measured the condensate composition as well as the sticking probability. When doing so, we see the same effect in the condensed mixed film, supporting an isotopic enrichment of the heavier isotope. We propose that enhanced multi-phonon interactions and inelastic cross sections between the incident CD_4_ projectile and the CH_4_ film allow for more efficacious gas–surface energy transfer. VENUS code MD simulations show the same sticking probability differences between isotopologues as observed in the gas–surface scattering experiments. Ongoing analyses of these trajectories will provide additional insights into energy and momentum transfer between the incident species and the interface. These results offer a new route for isotope enrichment via preferential condensation of heavier isotopes and isotopologues during gas–surface collisions under specifically selected substrate, gas-mixture, and incident velocity conditions. They also yield valuable insights into gaseous condensation under non-equilibrium conditions such as occur in aircraft flight in low-temperature environments. Moreover, these results can help to explain the increased abundance of deuterium in solar system planets and can be incorporated into astrophysical models of interstellar icy dust grain surface processes.

## Introduction

Adsorption is a key process in both astrophysical and terrestrial environments as it serves as the first step in many gas–surface interactions.^1−3^ In extraterrestrial environments where chemical species are scarce, adsorption onto an interstellar grain, planetesimal, or other larger body controls many combinatorial reactions. The formation of larger organic molecules becomes more feasible when species can engage on a surface rather than the void of space.^4−9^ In addition, the astrophysical environment is abundant with isotopes of many chemical species.^10^ In order to accurately model the chemical abundances, we need to better understand how differences in mass can influence the ability of a species to adsorb under specified conditions, and thus lead to observed relative isotope abundances.^11,12^

Interstellar methane is the most common hydrocarbon, existing in both the gaseous and the solid form.^13−19^ Methane is commonly found in the gaseous planetary atmospheres or as molecular ices intermixed with water ice matrices.^20,21^ As the most basic hydrocarbon, CH_4_ serves as a base for addition reactions which form larger hydrocarbon species.^22^ Additionally, the isotopic twin of CH_4_, CD_4_, can serve as a model for understanding the effects and the abundance of deuterium within these environments.^12,23^ Theoretical methods and gas chromatography have found that the isotopic difference in CH_4_ and CD_4_ stems from the difference in polarizability and length of the C–H and C–D bonds; however, no studies have reported how this difference might translate into its sticking probability.^12,24,25^ Studying CH_4_ and CD_4_ adsorption is an excellent model system to determine how slight mass differences in the condensate and projectile can impact adsorption and surface abundance of isotopic species.

Here we present the first study of the isotopic sticking probability of CH_4_ and CD_4_ as a function of translational beam energy on CH_4_ and CD_4_ thin films under ultrahigh-vacuum (UHV) conditions at low temperatures using the King and Wells method^26^ complemented by *in situ* infrared spectroscopic studies of gaseous condensation. VENUS code molecular dynamics (MD) simulations show the same sticking probability differences between isotopologues as were observed in the gas–surface scattering experiments. Taken together, these results accurately and independently determine the sticking probability, allowing us to explore how differences in isotopic composition of the surface and incident molecular mass can impact the overall energy accommodation, and thus adsorption of the gaseous species onto the film.

Key to these studies is the use of essentially monoenergetic seeded supersonic beams so that the CH_4_ and CD_4_ have the same incident velocity but different kinetic energies and momenta. It is shown that as the incident velocity of CH_4_ and CD_4_ increases, the sticking probabilities for both molecules on a CH_4_ condensed film decrease systematically, but that preferential sticking and condensation occur for CD_4_. These observations differ when condensed CD_4_ is used as the target interface, indicating that the film’s phonon and rovibrational densities of states, and collisional energy transfer cross sections, play a role in differential energy accommodation between isotopically substituted incident species. In addition, a mixture of gaseous CH_4_ and CD_4_ was grown on a methane thin film. While both species adsorbed creating a mixed isotopologue condensate, we saw an increased abundance of CD_4_ versus CH_4_ within the film as opposed to initial beam concentration. We demonstrate an isotopic enrichment for CD_4_ in our mixed surface based on the difference in sticking probabilities between CH_4_ and CD_4_.

This experiment builds on previous work by our group where CH_4_ sticking was investigated on the surfaces of D_2_O of varying morphologies and where H_2_O and D_2_O sticking on their own films were studied.^26−28^ In particular, we consider a similar isotopic experiment as the H_2_O and D_2_O sticking but expand to study CH_4_ and CD_4_ sticking on both films rather than only their own films.^27^ Additionally, previous work examined the sticking of only CH_4_ on H_2_O which we expand to include CH_4_ and CD_4_ sticking onto both CH_4_ and CD_4_ ices to examine how the mass difference can affect the overall sticking.^28^ We examine how these differences in mass, energy, and surface composition can affect the ability of the film to absorb and dissipate energy from the impinging molecules to allow adsorption onto the film structure.

Our work demonstrates differential condensation between methane isotopologues under specifically selected substrate, gas-mixture, and incident velocity conditions. The demonstrated outcomes have obvious implications for the development of novel isotopic enrichment and separation techniques. These results also provide new insights into gaseous condensation under non-equilibrium conditions such as occur in aircraft flight in low-temperature environments. More broadly, this work is critical to understanding the nature of methane adsorption within astrophysical environments. Our sticking probability differences can be incorporated into astrophysical models to explain molecular abundances and increased deuterium abundance in cometary ices and outer solar system planets. Aside from astrophysical environments, adsorption has implications into fields such as heterogeneous catalysis or thin film growth where the adsorption process serves as the first step in film formation.^28^

## Experimental Methods

All experiments were conducted in a molecular beam scattering instrument previously discussed in full detail.^28^ Briefly, this instrument consists of a UHV chamber with base pressures of 10^–10^ Torr connected to a triply differentially pumped molecular beamline. In the main chamber, a state-of-the-art closed-cycle helium-cooled sample manipulator (Advanced Research Systems) enables precise and accurate temperature control of the Au(111) sample substrate between 16 and 800 K. The crystal is exposed to the impinging beam at normal incident angle and monitored in real time with optics for *in situ* reflection absorption infrared spectroscopy (RAIRS). Gas scattering and incident flux monitoring occur with a residual gas analyzer (RGA).

All RAIR spectra are analyzed with Gaussian peak fitting atop cubic baselines. Spectra were acquired with a Nicolet 6700 infrared spectrophotometer (Thermo Fisher) using incident p-polarized IR radiation at an angle of 75° to the Au(111) crystal and a liquid-nitrogen-cooled mercury cadmium telluride (MCT/A) detector. Each RAIR spectrum is an average of 25–200 scans taken by using 4 cm^–1^ resolution with a clean Au(111) sample used for the background correction.

CH_4_ or CD_4_ was dosed on the Au(111) substrate via beam deposition at 18 K prior to measurements at 20 K. Dosing conditions resulted in a deposition rate of 0.5 layers per second.

CH_4_ and CD_4_ beams were produced by expanding 1% CH_4_ in H_2_ or 1% CD_4_ in H_2_ at stagnation pressures of 150–400 psi through a 15 μm platinum pinhole. Resistively heating the beam nozzle from room temperature up to 1100 K resulted in beam velocities of up to 4600 m/s and rotationally cold molecules resulting from the seeded expansion. The translational energy distribution widths (Δ*v*/*v*) ranged from 5 to 24%. We note that the velocity slip between the two isotopologues varied between 0 and at most 100 m/s with velocities spanning 2400 to 4600 m/s; therefore, the incident velocities of the two isotopologues were essentially identical for the purposes of a given experiment. Incident velocities were measured by time-of-flight methods using a mechanical chopper to modulate the beam prior to detection with an in-line quadrupole mass spectrometer. To confirm all the results and further understand phonon interactions at cold temperatures, a mixed beam was produced by expanding 1% CD_4_ and 3% CH_4_ in H_2_.

**Figure 1:**
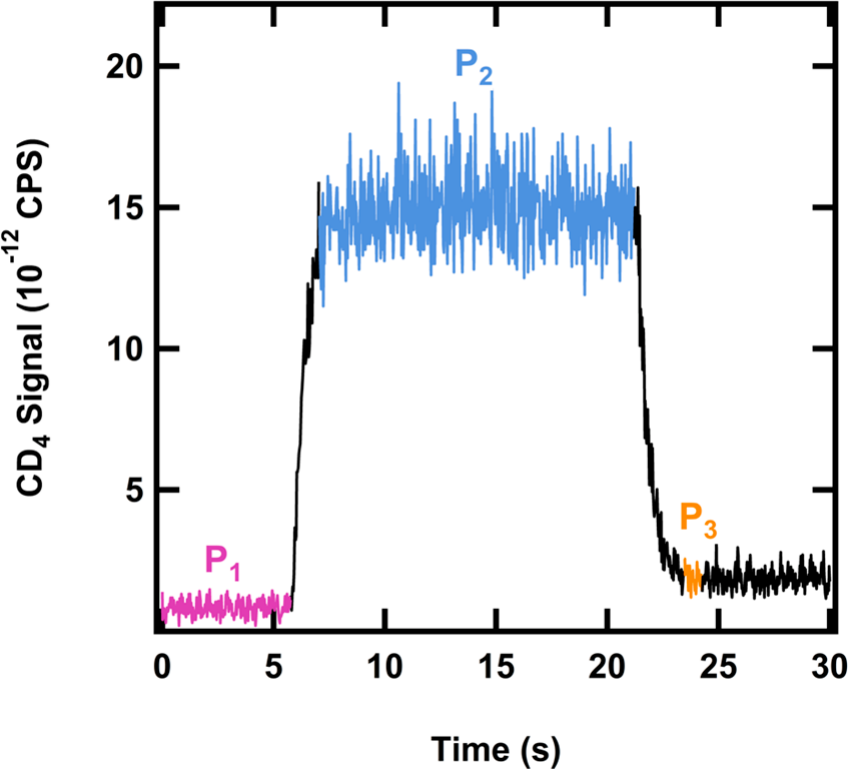
CD_4_ signal (*m*/*z* = 20) monitored with the RGA during a representative King and Wells experiment conducted on a CH_4_ surface at 20 K. *P*_1_ (pink) is the background CD_4_ signal, *P*_2_ (blue) is the full CD_4_ flux with the flag blocking the Au substrate, and *P*_3_ (orange) is the initial CD_4_ adsorption without the flag.

The sticking probability was determined by using the King and Wells technique.^26,29^ This was previously described in more detail for our system,^27^ and a typical King and Wells experiment conducted on a CH_4_ surface temperature at 20 K is shown in Figure 1 where *m*/*z* = 20 for CD_4_ is monitored as a function of time by using an RGA out of line with the beam. The experiment involves monitoring the background signal (*P*_1_), indirect flux (*P*_2_) with a flag in front of the substrate and removal of that flag where molecules start to stick (*P*_3_) to calculate the initial sticking probability:
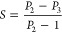


King and Wells measurements were performed at 20 K for all results presented in this study. This temperature was carefully chosen due to the methane surface interaction and the King and Wells method itself. UHV conditions at 20 K accurately model astrophysical chemistry rich environments such as dense molecular clouds.^1^ Additionally, at 20 K, multilayer CH_4_ is stable on a gold substrate and frozen ice films which enables measuring the condensate via RAIRS.^30,31^ As mentioned in He et al.,^32^ and detailed in our previous work examining the initial sticking probability of CH_4_ on D_2_O ices,^27^ the liquid helium cooling of the sample manipulator could impact the pumping speed and thus the reflected portion of the beam; therefore, we take all measurements at a single sample temperature. This ensures that the unknown pumping speed remains consistent across measurements. We also calculate sticking probability by using the initial CH_4_ indirect flux instead of the value at saturation.

## Results and Discussion

**Figure 2:**
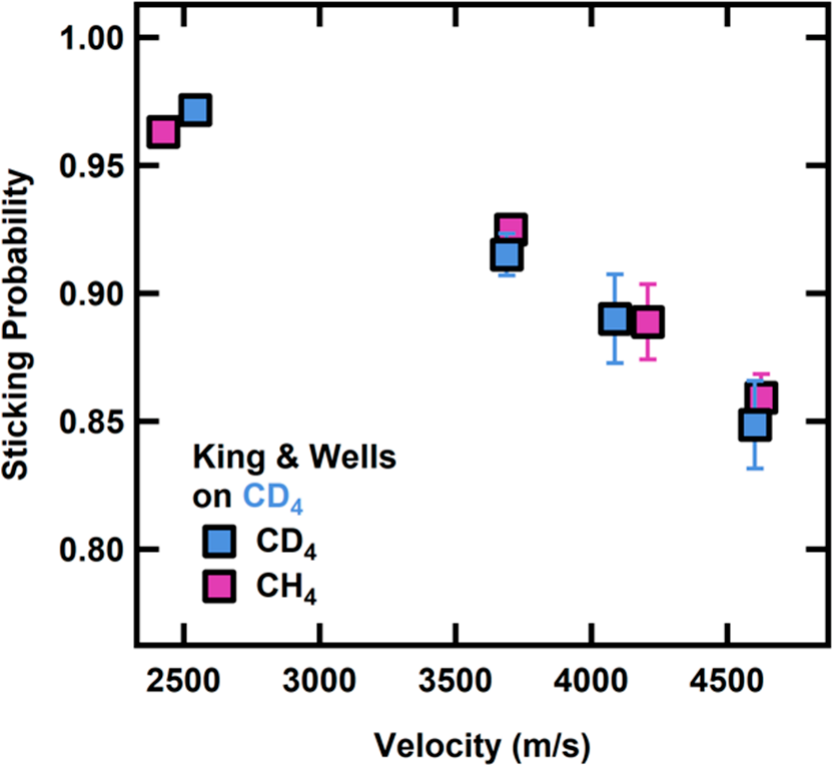
Sticking probabilities for CH_4_ and CD_4_ on a CD_4_ film at 20 K. The sticking probability decreases with increasing velocity. Error bars represent the standard deviation of at least three measurements on at least three different days.

To fully understand the role that mass matching and preadsorbed hydrocarbons play in trapping dynamics for CH_4_ and CD_4_, we examined sticking probability on top of amorphous CH_4_ and CD_4_. Although the sticking probability was previously found to be independent of ice film thickness,^33^ we choose to grow films for ∼80 layers to achieve self-similarity in film structure.^34−36^ The measured sticking probabilities for CH_4_ and CD_4_ on a CD_4_ substrate are shown in Figure 2. For physisorption trapping to occur, the CH_4_ or CD_4_ molecule must lose some initial kinetic energy when impinging upon the surface. If the energy loss is not efficient enough, the impactor molecule just bounces back. As expected, the sticking probability decreases with an increase in energy as more energy must be lost in the initial condensation in order for sticking to occur.^37,38^ The corrugation of the gas–surface potential for CH_4_ and CD_4_ is greater on the alkane-covered surface^39^ than it is on a bare metal substrate.^40,41^ Although our films are thicker than one monolayer, previous rare gas and alkene studies demonstrate that sticking probabilities are enhanced by such adlayers that allow for enhanced energy accommodation.^39,42^ Sticking probabilities are close to unity at low incident velocities for both incident isotopologues before decaying down to 0.85 for both CH_4_ and CD_4_. There was no strong variation in sticking probabilities between the CH_4_ and CD_4_ projectiles indicating, overall, very similar energy accommodation.^43^ This suggests that both phonon creation and translational to intramolecular energy transfer are essentially the same for both CH_4_ and CD_4_ on the condensed CD_4_ film.

**Figure 3:**
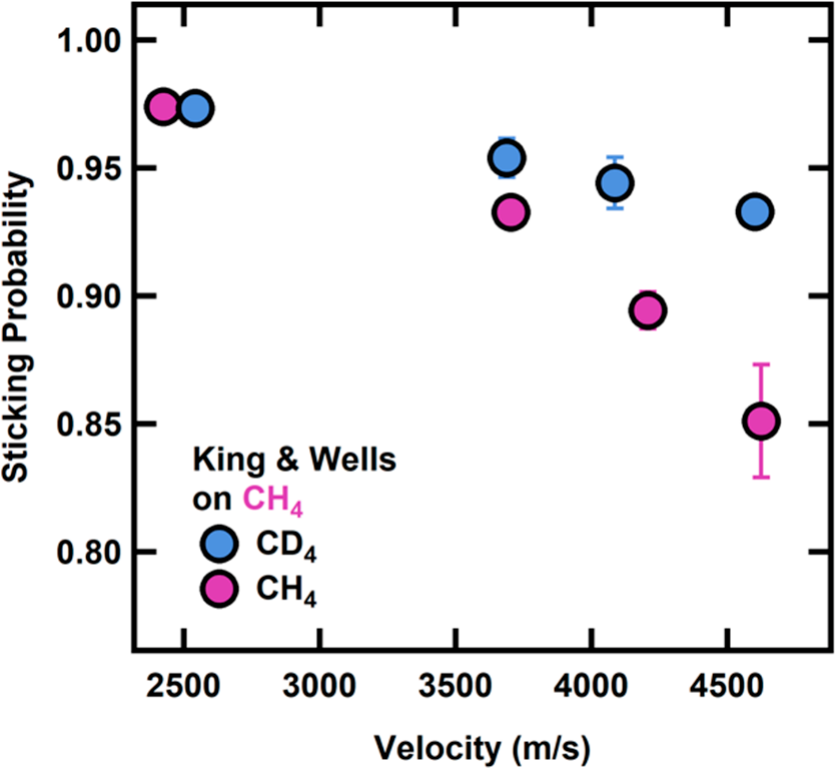
Sticking probabilities for CH_4_ and CD_4_ on a CH_4_ film at 20 K. The sticking probability decreases with increasing velocity, but remains higher for the CD_4_ projectile. Error bars represent the standard deviation of at least three measurements on at least three different days.

**Figure 4:**
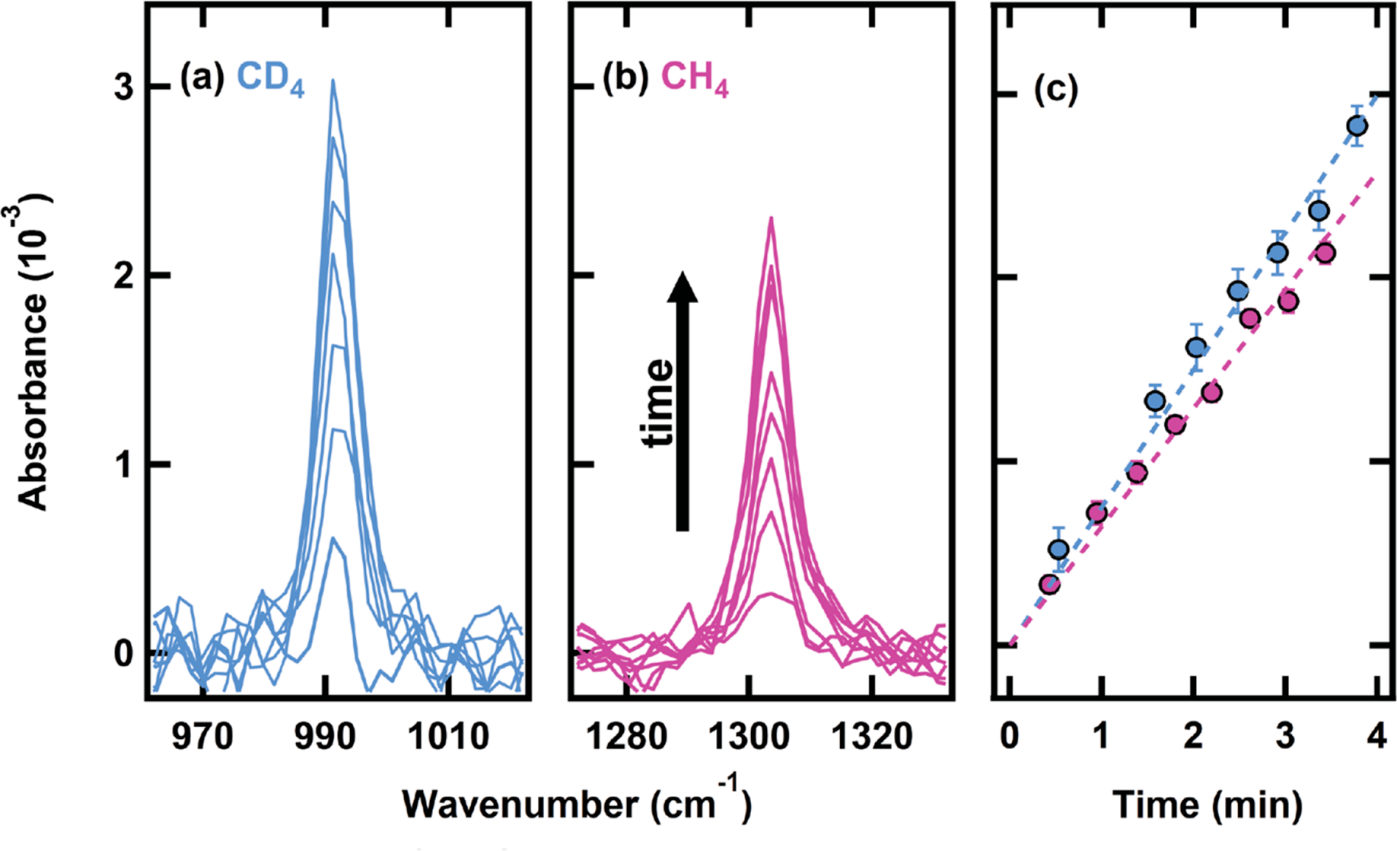
Representative RAIR spectra of CD_4_ (a) and CH_4_ (b) ν_4_ bending mode as a function of exposure time for the highest energy beam (4600 m/s) on a CH_4_ surface at 20 K. Spectra were taken approximately every 25 s (c) as a function of intensity to get the initial growth rate. Differences in sticking probability result in an increased amount of CD_4_ on the surface and thus a higher growth rate.

*However*, *we note a higher sticking coefficient for CD*_4_*on CH*_4_*ice than for CH*_4_*on CH*_4_*ice particularly at high incident translational energies* (Figure 3). We monitored the amount of adsorbed CH_4_ and CD_4_ via the intensity of the degenerate ν_4_ bending mode^44−48^ to calculate the initial growth rate. To ensure that these measurements were taken during an essentially constant film thickness regime, the IR measurements were completed by adding no more than an additional 0.75 MLs of condensate over <4 min. As shown in Figure 4 for CH_4_ and CD_4_ beams at 4600 m/s, sticking probability differences between the CH_4_ and CD_4_ result in a larger amount of CD_4_ stuck on the surface after exposure and therefore a higher initial growth rate. Based on the total spectral intensity vs time and, thus, condensed projectile on the surface, we calculated the initial growth rate for each incident velocity. As a consistency check, at the end of the growth exposure, we took an additional King and Wells measurement, which matched the initial sticking probability at the beginning. Taken together, this indicates that the coverage following the growth rate is not enough to change the underlying film structure and that RAIRS allows us to determine the amount of CH_4_ or CD_4_ on the surface. When overlaying this with the initial sticking probability (Figure 4), we confirm that both the King and Wells measurements and infrared spectroscopy of the condensate demonstrate an increased condensation of CD_4_ on CH_4_ compared to CH_4_ on CH_4_.

**Figure 5:**
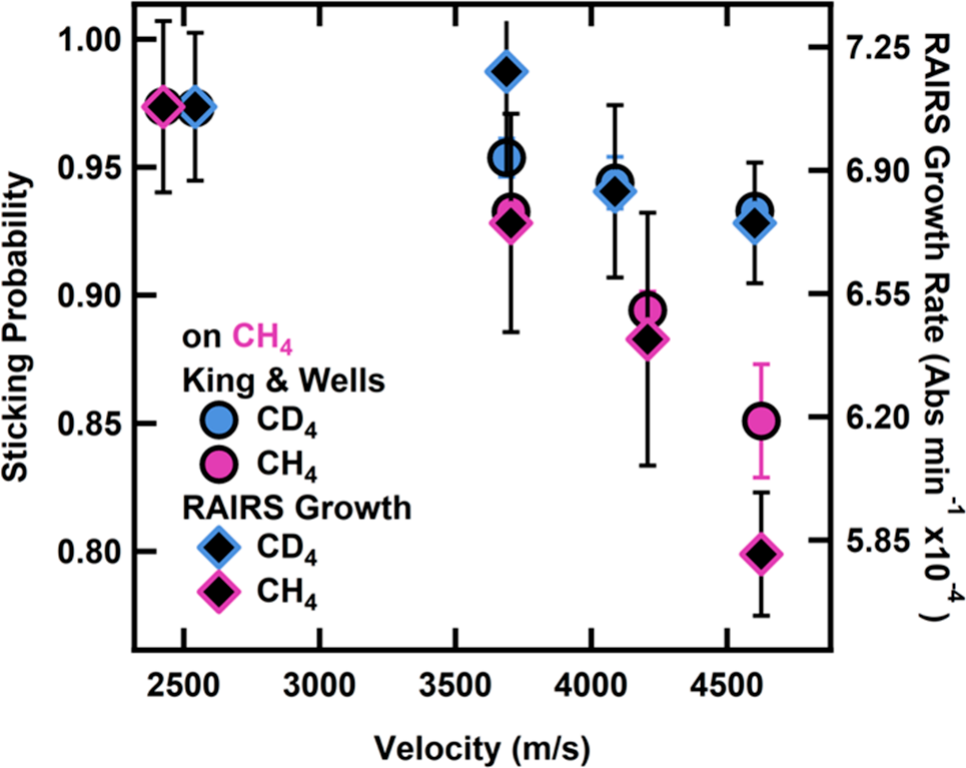
Confirmation of increased condensation of CD_4_ on CH_4_ in comparison with CH_4_ on CH_4_ as a function of incident methane velocity. Monitoring of the amount of adsorbed methane via the intensity of the ν_4_ bending mode for CH_4_ and CD_4_ by RAIRS, we calculate the initial growth rate to overlay with the sticking probability. Error bars represent the standard deviation of at least three measurements on at least three different days.

This isotopic effect increases with increasing translational energy. To understand this, we start by examining the Baule model,^49^ which predicts that a more efficient collision occurs when the gas and surface masses match due to the singularity in the momentum case. An incident molecule containing mass *m* and energy *E* encounters a square well potential of depth *D* and a surface species of mass *M*, resulting in an energy transfer (Δ) in the collision:^40^
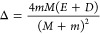
For this, we assume that the energy of the incoming molecule is much greater than the well depth of the potential. Thus, for the CH_4_ film, the energy transfer for the CD_4_ projectile is greater than that of the CH_4_, which would generally indicate a higher sticking probability. This model does not account for the density of states of the film or the internal modes of the molecule, as discussed later. These contributions can influence sticking probabilities.^50,51^ Because of the role of these molecular degrees of freedom, complex and multi-phonon interactions^52,53^ between the surface and the incident projectiles CH_4_ film and CD_4_ clearly need to be taken into consideration, as they are in the MD simulations shown herein.

We performed chemical dynamics simulations using the VENUS MD computer program.^54,55^ Classical trajectories simulated collisions of a beam of CH_4_ or CD_4_ with the CH_4_ or CD_4_ surface at a surface temperature of 20 K. Initial conditions for the trajectories were selected to sample the experimental beam’s translational and vibrational energy. After collision the trajectories were terminated at 50 ps; CH_4_ and CD_4_ remaining on the surface were considered trapped. The scattered trajectories are dominated at the level of ∼99% by direct scattering rather than those that trap and then desorb.

## Computational Details

The potential energy function for the (CH_4_)_beam_ and (CH_4_)_surface_ on top of a Au(111) crystal is given by
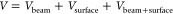
where *V*_beam_ is the beam CH_4_ intramolecular potential. *V*_surface_ is composed of intramolecular CH_4_ potentials (same as *V*_beam_) as well as the intermolecular CH_4_---CH_4_ and Au---CH_4_ potentials using the 6–12 Lennard-Jones fashion. Lastly, *V*_beam+surface_ is the intermolecular CH_4_---CH_4_ potential. Each intramolecular CH_4_ potential is expressed as a sum of Morse potentials for the C–H stretches and quadratic potentials for the H–C–H bends: the Morse parameters are *D* = 112.5 kcal/mol, β = 1.86 Å^–1^, and *r*_0_ = 1.086 Å,^56^ and each HCH quadratic bend has *f* = 0.585 mdyn Å/rad^2^ and θ = 109.47°.^56,57^ The methane harmonic frequencies are 3193, 3021, 1583, and 1413 cm^–1^.

The surface model consists of six methane layers stacked in an AB sequence on top of a layer of gold to form a cubic close-packed structure.^58^ There are 789 CH_4_ molecules in alternating layers of 120/143 molecules to so that *x* and *y* are each 40 Å for an area of 800 Å for each layer. The total surface height of all the stacked layers is 18 Å, including the gold layer on the bottom. All intermolecular potentials are written as sums of Lennard-Jones two body potentials with a cutoff distance of 10 Å and are summarized in Table 1. For the Au (111) base, ε_0_ = 5.29 kcal/mol and σ_0_ = 2.951 Å^59^ were used to give an atomic spacing of 2.93 Å, closely matching that determined from STM images of the reconstructed (111) surface.^60^ Our surface contains CH_4_ spaced by 3.8 Å, which is comparable to calculated CH_4_ intermolecular potentials.^61^ CH_4_---CH_4_ intermolecular potentials among all methane molecules (including those in different layers) are written as sums of 6–12 Lennard-Jones two-body potentials and include interactions between carbons and hydrogens.^62,63^ To calculate the Au---CH_4_ interaction, we employ standard mixing rules^59,64^ and assume a geometric mean between C and Au to get a ε_0_ = 0.7336 kcal/mol and σ_0_ = 2.99 Å. Geometry optimization of the surface occurred prior to trajectory simulations to obtain a potential energy minima configuration. Additionally, we note that this is a flat crystalline surface, which a model representation of a local section in the experimental surface topology which in reality may contain domains of small, imperfect crystallites. However, even with this difference, there is qualitatively similar energy-transfer dynamics and thus is appropriate to use for our study.^65^

**Table 1:** Parameters of the Lennard-Jones 12–6 Atom–Atom Interactions

	ε_0_ (kcal/mol)	σ_0_ (Å)
Au–Au	5.29	2.951
C–C	0.1017	3.35
C–H	0.0473	2.99
H–H	0.0097	2.61
Au–C	0.7337	2.99
Au–H	0.0	0.0

A microcanonical ensemble averaged intermolecular potential curve for CH_4_ approaching to the surface is obtained by averaging the potential energies of randomly oriented CH_4_ as a function of CH_4_-surface center-of-mass separation parallel to the surface norm.^66^ Such a potential energy minimum is −0.07 eV at a center-of-mass separation of 4.25 Å.

### Procedure for the Chemical Dynamics Simulations

Chemical dynamics simulations were performed by using the VENUS general chemical dynamics computer program.^54,55^ Classical trajectories were used to simulate collisions of a beam of CH_4_ or CD_4_ with the CH_4_ or CD_4_ surface. Simulations at each collision energy were performed by using a surface temperature of 20 K. Initial conditions for the trajectories were selected to sample the beam’s translational and vibrational energy at the experimental conditions. The selection of initial conditions follows from previous VENUS studies.^67,68^ For each simulation, a beam of colliding molecules was aimed within a circular area. Each trajectory was initialized with a separation of 10 Å between the center of the beam and surface aiming point. For each beam, the initial vibrational quantum states were sampled from Boltzmann distributions at 300, 700, 900, or 1100 K, and the translational energies were determined from the molecular beam velocity distributions (Figure 2). Using the experimental velocities, the CH_4_ translational energies were 0.49, 1.16, 1.48, and 1.79 eV and the CD_4_ translation energies were 0.67, 1.41, 1.74, and 2.19 eV. Zero-point energy was included in these samplings, and the rotational energy was set to 0 K to match the experimental supersonic molecular beam conditions.

For each trajectory, the gold and bottom three layers were held rigid and acted as anchor layers. Additionally, the mass of carbon atoms in rim CH_4_ molecules was artificially increased by 10000 to truncate the surface. Initial conditions for this surface were selected by assigning velocities to the carbon atoms of these layers, sampled from a Maxwell–Boltzmann distribution at 20 K. The surface was equilibrated by a 50 ps molecular dynamics simulation with velocity scaling every 1000 steps and another equilibration without velocity scaling. The trajectories were propagated with a Velocity-Verlet integrator, with a time step of 0.01 fs. Trajectories were terminated when either the distance between the central methane molecule and outgoing product exceeds 30 Å or the total integration exceeds 50 ps. Typically, 750–2000 trajectories were calculated for each ensemble of initial conditions including the surface composition and beam conditions.

### Simulation Results

**Figure 6:**
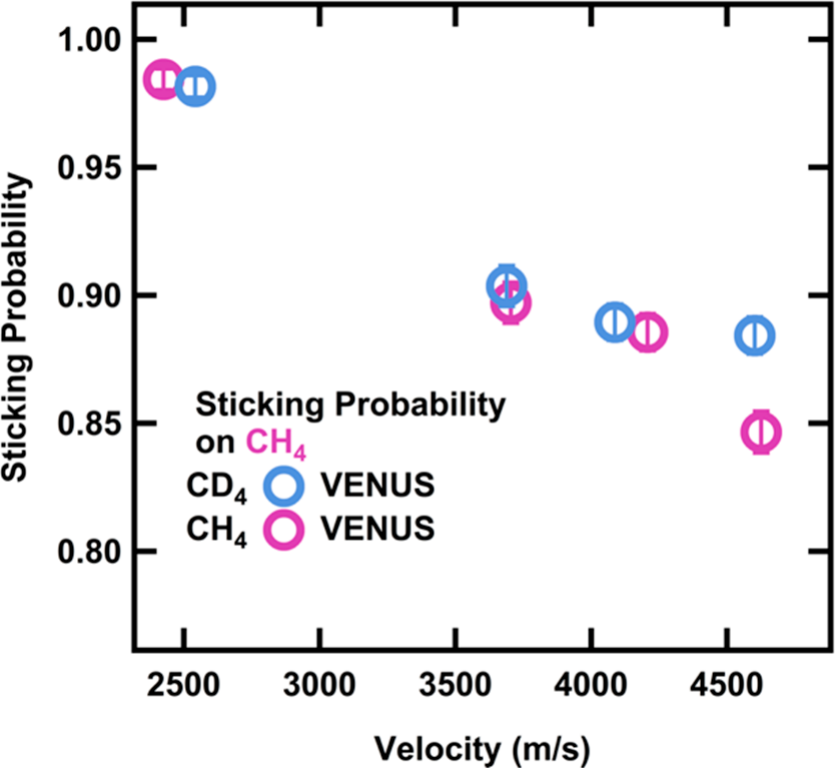
Sticking probabilities calculated from the number of CH_4_ and CD_4_ direct and physisorption scattering trajectories on a CH_4_ layered surface at 20 K. Error bars represent the standard error of at least 750 trajectories for each velocity.

**Figure 7:**
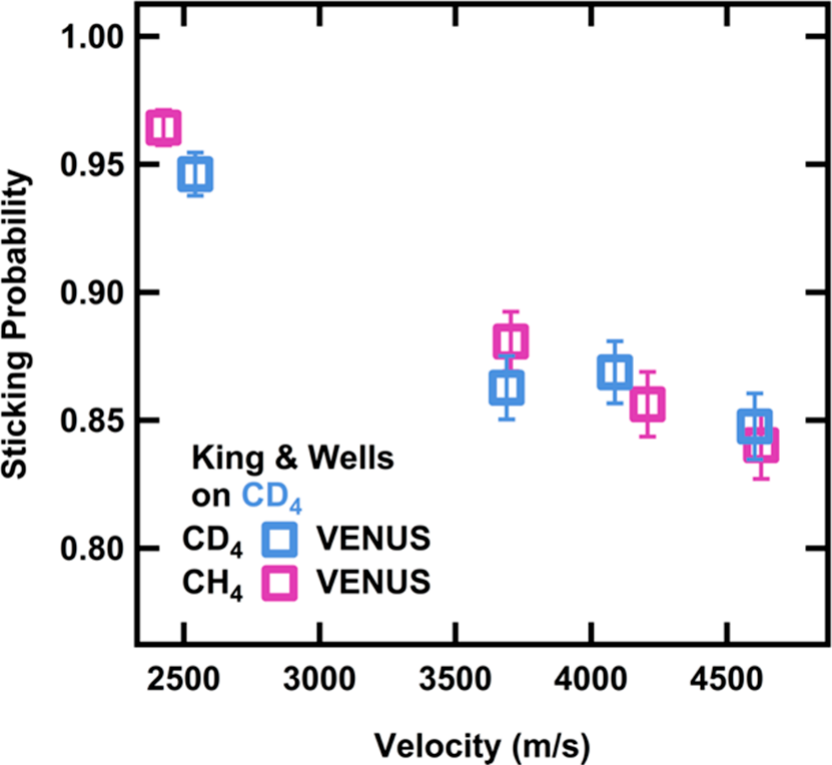
Sticking probabilities calculated from the number of CH_4_ and CD_4_ direct and physisorption scattering trajectories on a CD_4_ layered surface at 20 K. Error bars represent the standard error of at least 750 trajectories for velocity.

Overall, we find that there is nice agreement between the chemical trajectory simulation results and the experimentally determined sticking probabilities. The VENUS calculations demonstrate a decrease in sticking probability with increasing incident velocity as well as a difference between CH_4_ and CD_4_ on a CH_4_ surface (Figure 6). Additionally, for the simulated collisions on a CD_4_ surface (Figure 7), there is no difference in sticking probability, again in agreement with our experimental results. In a more careful comparison to the results shown in Figure 3, the theoretical sticking probability for the CD_4_ on the CH_4_ surface is slightly lower than the experimental value. This could arise from various effects; for example, Lennard-Jones potentials are not optimized for the repulsive region.^69^

Full details of the energy transfer and chemical dynamics simulations will be discussed in a forthcoming paper to provide a molecular-level understanding of the mechanisms occurring between the methane projectile and the methane surface. When examining phonon dispersion curves for CH_4_ and CD_4_,^70^ not only are the CD_4_ phonon modes at a lower energy, but there is enhanced translational–rotational coupling.^71^ In addition to this coupling, local corrugation of the surface can also influence trajectory paths and therefore energy flow.^72,73^ Full analysis of our molecular dynamics studies will provide necessary insight into lattice vibrations and how energy is efficiently dissipated to trap the methane isotopologues.

**Figure 8:**
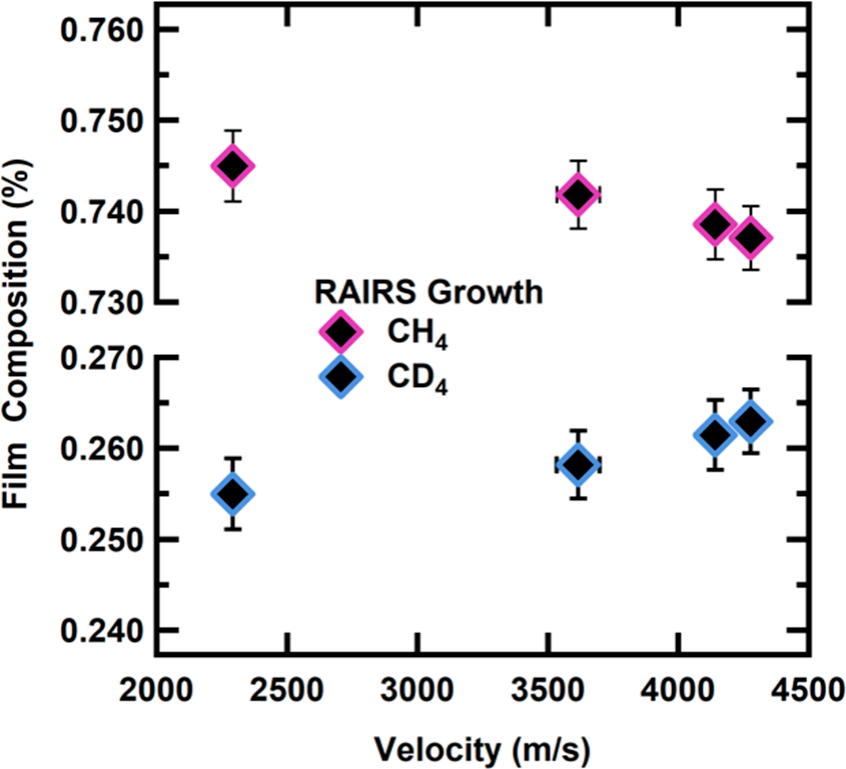
Integrated area of the ν_4_ mode for CD_4_ and CH_4_ demonstrates an enrichment of the heavier isotope (CD_4_) into the mixed condensed film at higher beam velocities. Error bars represent the standard deviation of at least 35 steady-state films on at least three different days.

To further explore and confirm our experimental results, we consider a beam composed of both CH_4_ and CD_4_ in a 3:1 ratio; this ratio was not selected to optimize condensation differences, but rather to demonstrate the robust nature of differential sticking. This allows us to quantify the sticking probability as well as condensate composition. While dosing a multilayer film of both CH_4_ and CD_4_ at 20 K, the integrated area of the degenerate ν_4_ mode was tracked over time using RAIRS.^44−48^ Once the condensate reached a self-similar structural steady state of at least 100 layers, at least 10 spectra per experiment on at least three different days were averaged to determine the film composition. As depicted in Figure 8, the condensate composition for the room temperature beam (2200 m/s) is 74.5% CH_4_ and 25.5% CD_4_. However, as the beam velocities increase, the heavier isotope (CD_4_) becomes preferentially adsorbed into the film. Because of increased adsorption into the film, the condensate film structure changed to 73.7% CH_4_ and 23.6% CD_4_. *Overall, by measuring the condensate with RAIRS, we confirm that due to the increased sticking probability of CD*_4_*on a CH*_4_*film, we see an increased affinity for CD*_4_. We demonstrate for our fastest beam (4400 m/s) that there is a 3.12 ± 0.06% enrichment of CD_4_ compared to the room temperature beam (2200 m/s). When taking the individual King and Wells values (Figures 2 and 3) and combining that with the film composition determined from the RAIR spectra, we calculate the sticking probabilities for CH_4_ and CD_4_ on the mixed film. For the highest velocity beam, these sticking probabilities also result in a CD_4_ enrichment of 3.9 ± 0.02%, indicating excellent agreement with the observed condensate enrichment.

## Conclusion

We examined the differential sticking probability of CH_4_ and CD_4_ on CH_4_ and CD_4_ ices using RAIRS for measuring on-surface gaseous condensation and complementary King and Wells mass spectrometry techniques for monitoring sticking probabilities. We found that as the incident velocity of CH_4_ and CD_4_ increases (up to 4600 m/s), the sticking probability decreases for both films. Interestingly, we conclude that preferential sticking and condensation occurs for CD_4_ when striking the surface in comparison to the outcome for CH_4_. This observation was confirmed both experimentally from infrared spectroscopy of the condensation and via mass spectrometric detection of the reflected molecules and theoretically from the gas–surface chemical trajectory simulations. This theoretical model system will be explored in more detail to provide insight into energy transfer and lattice vibrations. Next, we employed a mixed incident supersonic beam composed of both CH_4_ and CD_4_ in a 3:1 ratio to measure the condensate as well as the sticking probability. When doing so, we see the same effect in the condensed mixed film, supporting an isotopic enrichment of the heavier isotope. Because the Baule model^74^ does not accurately represent this condensed phase system due to its molecular complexity, we propose that enhanced multi-phonon interactions attributable to the film’s phonon and rovibrational densities of states and inelastic cross sections including intermolecular energy exchange between the incident CD_4_ projectile and the CH_4_ film allow for more efficacious gas–surface energy transfer.

In general, these results indicate the importance of understanding gas–surface energy exchange under non-equilibrium conditions at cold substrate temperatures and have important astrophysical and terrestrial implications. Our work demonstrates the importance of film structure and surface lattice coupling to allow for efficient energy transfer and an isotopic enrichment of the heavier isotope (CD_4_) The insights gained from gaseous condensation under nonequilibrium conditions are also important for understanding aircraft flight in low-temperature environments. These results also offer a new route for isotope enrichment via the preferential condensation of heavier isotopes and isotopologues during gas–surface collisions under carefully selected substrate, gas-mixture, and incident velocity conditions.

Importantly, our experiments are conducted at low-temperature astrophysical conditions. By experimentally determining initial sticking probability differences between methane and its heavier isotopologue as a function of incident energy, we find that the film composition is important, especially for high-energy projectiles bombarding icy dust grains. Because adsorption is often a first step for many cold temperature reactions occurring on these grains, differences in sticking probabilities have notable implications for allowed reaction probabilities and follow-on events leading to increased molecular complexity. Our work, therefore, not only can explain increased abundance of deuterium in solar system planets^75,76^ but also can be incorporated into astrophysical models of the icy dust grain processes including those in the interstellar region.^2^
